# A case of combined cranial nerve palsy after general anesthesia

**DOI:** 10.1186/s40981-018-0211-0

**Published:** 2018-10-08

**Authors:** Chiho Uneda, Toshiyuki Yano, Takashi Imaizumi

**Affiliations:** Division of Anesthesia, Kumamoto Kinoh Hospital, 6-8-1 Yamamuro, Kita-ku, Kumamoto, 860-8518 Japan

**Keywords:** Complication, Hypoglossal nerve, Neurapraxia, Recurrent laryngeal nerve, Tapia’s syndrome

## To the editor

We encountered concomitant paralysis of the unilateral vocal cord and tongue after upper extremity surgery under general anesthesia. This presentation is very rare but may be clinically instructive for both anesthesiologists and surgeons.

## Case presentation

A 51-year-old man (165 cm, 81 kg) underwent internal fixation of a left proximal humeral fracture under general anesthesia. After inducing anesthesia with propofol, remifentanil, and rocuronium, an 8.0-mm tracheal tube was placed using a Macintosh blade uneventfully. The depth and intracuff pressure of the tracheal tube were 23 cm and ≤ 12 cmH_2_O, respectively. The 105-min long procedure was performed in the beach-chair position. Anesthesia was uneventful, except for the displacement of the patient’s head and neck, which required repositioning—the head and neck were often deflected to the right while traction was applied to the left forearm during fracture reduction. After emergence from anesthesia, the patient was hoarse. On postoperative day 1, he complained of dysarthria, dysphagia, and leftward deviation of the tongue. On postoperative day 2, magnetic resonance imaging of the head did not show any abnormalities. Subsequent swallowing videofluoroscopy and laryngeal endoscopy revealed pharyngeal retention without aspiration and left vocal cord paralysis. He was diagnosed with combined left hypoglossal and recurrent laryngeal nerve palsy. All symptoms subsided within 8 months, without systemic administration of corticosteroids.

## Discussion

**Fig. 1:**
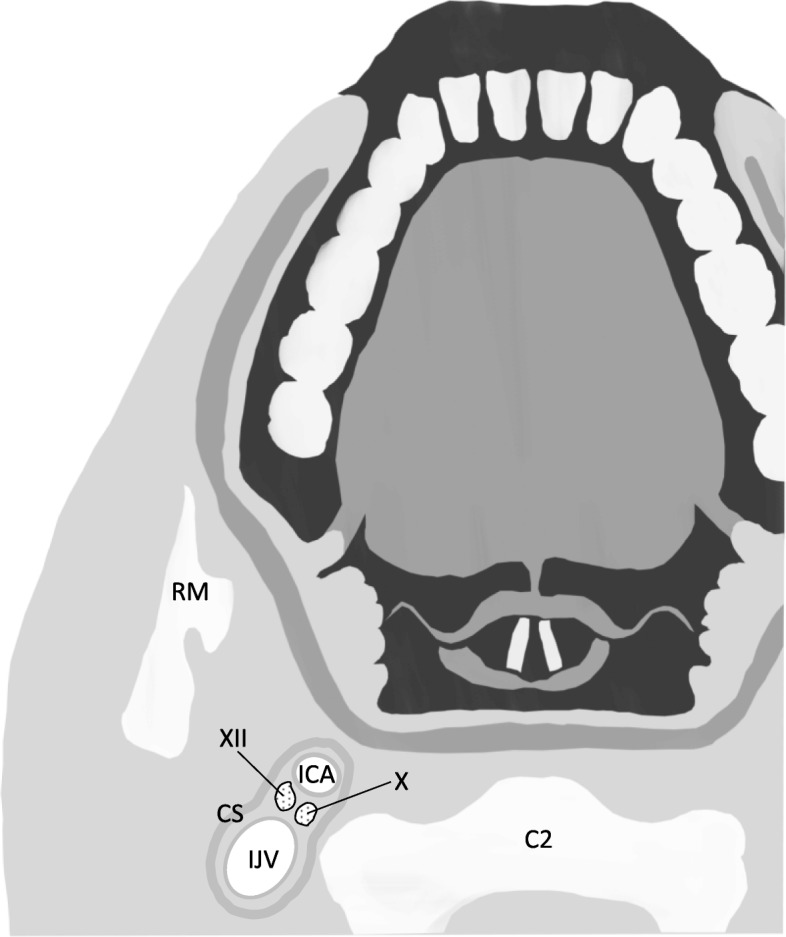
A sectional illustration of the oropharynx at the level of the second cervical vertebral body (C2). Note that cranial nerves X and XII are adjacent to the carotid sheath (CS) and are located immediately behind the mucosal surface of the lateral oropharyngeal wall. RM ramus of the mandible, IJV internal jugular vein, ICA internal carotid artery

Combined unilateral palsy involving extracranial lesion of cranial nerves (CN) X and XII is known as Tapia’s syndrome; this is primarily caused by carcinoma, inflammation, or injuries and rarely occurs after general anesthesia [1, 2]. Mechanisms underlying anesthesia-related Tapia’s syndrome remain unclear. Several plausible explanations involve the path of CN X and XII in the lateral wall of the orohypopharynx, where these two nerves run in parallel, near the mucosa (Fig. 1) [3–5]. CN X and XII in this region might be vulnerable to direct compression by oropharyngeal instrumentation, including a laryngoscope blade, tracheal tube, supraglottic airway device, transesophageal echocardiography probe, or throat pack [1, 3–10]. Excessive head and neck displacement during airway manipulation and surgery might cause malpositioning of these instruments; it might also stretch CN X and XII against the transverse process of the first cervical vertebra, as these nerves cross each other near this process [1, 4, 5, 7–13]. Compression and/or stretching of CN X and XII could cause neurapraxia [1–3, 5, 9]. Concomitant CN X and XII neurapraxia could occur at anatomically separate sites, owing to compression by oropharyngeal instrumentation [1, 2, 8–10]. Most patients recover from neurological deficits, but some may require corticosteroid therapy [2, 7, 12]. Anesthesiologists and surgeons should consider the possibility of combined CN X and XII palsy occurring in various procedures not involving direct access to these nerves [7, 12]. Communication between anesthesiologists and surgeons about posture may prevent this complication. Moreover, close attention should be given to coexisting symptoms in patients with hoarseness, a common complication of general anesthesia; this will ensure the accurate and timely diagnosis of Tapia’s syndrome, possibly preventing poor recovery or a life-threatening outcome, such as aspiration [4, 11, 14].

## Abbreviations

CN: Cranial nerve
